# Protein evolution of ANTP and PRD homeobox genes

**DOI:** 10.1186/1471-2148-8-200

**Published:** 2008-07-11

**Authors:** Nuno A Fonseca, Cristina P Vieira, Peter WH Holland, Jorge Vieira

**Affiliations:** 1 Instituto de Biologia Molecular e Celular (IBMC); University of Porto, Rua do Campo Alegre 823, 4150-180 Porto, Portugal; 2 Department of Zoology, University of Oxford, South Parks Road, Oxford, OX1 3PS, UK

## Abstract

**Background:**

Although homeobox genes have been the subject of many studies, little is known about the main amino acid changes that occurred early in the evolution of genes belonging to different classes.

**Results:**

In this study, we report a method for the fast and efficient retrieval of sequences belonging to the ANTP (HOXL and NKL) and PRD classes. Furthermore, we look for diagnostic amino acid residues that can be used to distinguish HOXL, NKL and PRD genes.

**Conclusion:**

The reported protein features will facilitate the robust classification of homeobox genes from newly sequenced bilaterian genomes. Nevertheless, in non-bilaterian genomes our findings must be cautiously applied. In principle, as long as a good manually curated data set is available the approach here described can be applied to non-bilaterian organisms as well. Our results help focus experimental studies onto investigating the biochemical functions of key homeodomain residues in different gene classes.

## Background

Genes that belong to the homeobox family are characterized by the ability to code for a protein that contains a recognizable, although very variable, 'homeodomain', usually 60 amino acids in length [1,2]. Many of these genes are transcription factors that play important roles in the embryonic development of bilaterian and non-bilaterian animals. Changes in homeobox gene content and deployment during evolution may have contributed to the evolution of body plan differences in animals [3-5]. Therefore, comparison of homeobox gene sets from different animals may shed light on the evolutionary events that gave rise to animal body plan diversity. Nevertheless, gene orthology is not always easy to establish when comparing divergent animals [6]. For this purpose, phylogenetic analysis, conservation of synteny, paralogy within the human genome, insertions within the homeodomain, key amino acid residues, and several motifs outside of the homeodomain can all be used. These features can also be used to classify homeobox genes into classes, subclasses and families. In the latest revision, Holland et al. [6] classified all 235 human homeobox genes into 11 classes (ANTP, PRD, LIM, POU, HNF, SINE, TALE, CUT, PROS, ZF and CERS) and 102 gene families. The ANTP class is further divided into HOXL and NKL subclasses. It should be noted that the only protein region that can be aligned in all 102 gene families or 235 genes is the homeodomain [6].

Here, we report amino acid patterns typical of bilaterian HOXL, NKL, and PRD genes that can be used to quickly and efficiently retrieve amino acid sequences belonging to these classes and subclasses, among hundreds of other homeodomain sequences. Retrieving a given class or subclass of sequences from many animal genomes may be thus an easier task than previously thought. However, we show that these typical amino acid patterns should be cautiously used when sequences come from non-bilaterian animals.

Since phylogenetic analysis was one of the primary sources of evidence used to establish the different classes and sub-classes, it is likely that most of homeobox gene classes and subclasses represent monophyletic lineages. Hence, it is conceivable that amino acid changes important for protein function in a given lineage may be revealed as fixed differences between classes and subclasses. Previous attempts have been made to classify genes within HOX families [7-9] but not at the level of whole classes or subclasses. Here, we show that, because of their chemical properties, amino acid usage is different in ANTP and PRD classes at five positions. Furthermore, at nine positions, amino acid usage is different between HOXL and NKL subclasses. Our findings support the notion that many chemically important changes happened early in the evolution of homeobox genes, and that these changes can be used as additional evidence to establish gene orthology. These results can be helpful for experimental studies aimed onto investigating the biochemical functions of key homeodomain residues in different gene classes as well.

## Methods

### Data

We have used the hand-curated data set of Holland et al. [6], the PROSITE homeodomain data set [10] and the NCBI database [11].

### Identification of amino acid patterns typical of HOXL, NKL and PRD genes

In order to find amino acid patterns that distinguish genes from the ANTP subclasses, as well as genes from the PRD class, from genes belonging to other classes, a fast word discovery program ([12]; available at [13]) was first used to find statistically interesting words. The minimum word length used was two (-m 2) and the minimum number of sequences where the word should occur (-e) was set to 10% of the number of sequences in the data set being considered. The words found were then filtered to discard those words that occurred more than three times in more than one dataset. The software Bioredx ([14]; available at [13]) was then used to find the largest and more general amino acid patterns that distinguish two sets of sequences. This program accepts as input two sequence files and a word seed. Based on the seed given, tries to find patterns that occur in one file (referred as the positive file) and not in the other (referred as the negative file). This software was used to find patterns that occurred less than four times in the negative file and considered patterns up to 20 amino acid residues larger than the initial word seed.

In the reported amino acid patterns, amino acids listed within brackets are those allowed at a given position. For compactness of representation it is also possible to negate the class. The negation is denoted by "^". In this case, the amino acid residues listed are the ones that are not allowed at that particular position. The approach here used to find amino acid patterns does not use a set of aligned sequences. Therefore, in order to make sure that the derived amino acid patterns are found in the same region of the homeodomain sequence, all sequences belonging to a given class and sub-classes were aligned using the PAM 250 scoring matrix [15].

### Identification of key amino acid residues

There are many schemes for comparing and grouping amino acids. Nevertheless, none of them can possibly capture the vast number of contexts in which amino acids are found within proteins. The scheme here used is that of Livingstone and Barton [16], based on the amino acid properties size, polarity, hydrophobicity, charge, aliphaticity and aromaticity. For each position and property we calculated the probability of gaining or losing a constraint using the distribution of the amino acid property's observed at that position in all sequences considered. Only changes in the properties values with a probability of occurrence lower than 5% were considered.

### Phylogenetic analyses

A Neighbour-joining tree, using pair-wise deletion, as implemented in the MEGA software [17] was constructed in order to classify the set of 918 homeodomain sequences from a variety of animal species (available at PROSITE [10]), into the 12 classes and two sub-classes scheme proposed by Holland et al. [6].

## Results

In order to establish a method for fast retrieval of sequences belonging to a given class or subclass, amino acid patterns characteristic of different homeobox classes and subclasses were sought. As a starting point we used the hand-curated human PRD, NKL and HOXL data sets compiled by Holland et al. [6]. The homeodomains of human EN1, EN2, DLX1, DLX2, DLX3, DLX4, DLX5, DLX6, NOTO, and HOPX are difficult to classify and were not used in these initial analyses. Therefore, the PRD, NKL and HOXL data sets contain 49, 39 and 52 sequences, respectively. Within each of the three data sets, the amino acid sequences show considerable variation, suggesting that the human sequences include the majority of amino acid variability allowed at a given position along the sequence. This assumption was later tested (see section 2).

### 1) Amino acid patterns characteristic of the different human gene classes and sub-classes

Many characteristic amino acid patterns were detected using the approach described in Material and Methods. Here, we describe the seven patterns that are most pertinent, taking into account their coverage across a group of genes and absence in the other groups.

**Figure 1:**
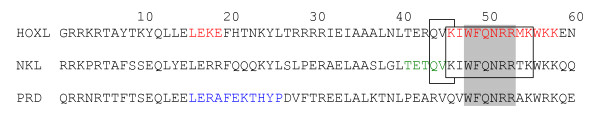
**Majority rule consensus sequence of HOXL, NKL and PRD genes**. The relative location of the described amino acid patterns is shown (see text for details). Red – HOXL1 and HOXL2 patterns. Green – NKL pattern; Blue – PRD pattern; Boxed – ANTP1 and ANTP2 patterns; Grey shadow – ANTP-PRD pattern.

The pattern [KT] [IV]WFQNRR [AMV]K [DEHKLMQWY] [KR] [KR] (positions 46–58; Fig. 1), named HOXL 1 pattern, is present in all human HOXL homeodomains, and in none of the human NKL and PRD genes.

The pattern LE [AGKNR]E (positions 16–19; Fig. 1), named HOXL 2 pattern, is present in all human HOXL homeodomains except *Mnx1*. Only two human PRD genes (*Pax4 *and *Pax6*) encode this amino acid pattern. None of the human NKL genes encode this amino acid pattern.

The pattern [AKST] [DENPS] [LAST] [Q] [V] (positions 41–45; Fig. 1), named NKL pattern, is present in 36 out of 39 human NKL sequences, in one human HOXL sequence (Mnx1), and in none of the human PRD sequences. The human NKL sequences that do not present this pattern are: HHEX, NANOG, and VENTX.

The pattern L [EINQRV] [^DGHMPTVWY] [^CDGKMNPQR] [FL] [^CFILPTWY] [AEFHKQRV] [ADEGKNSTW] [CHKMPQR] [FHY]P (positions 16–26; Fig. 1), named PRD pattern, is present in all human PRD homeodomains and never present in human HOXL and NKL sequences.

The patterns [HQ] [IV] [AKLT] (positions 44–46; Fig. 1), named ANTP 1 pattern, and [IKTV] [ITV]W [FY]QN [HQR]R [AMNTVY]K, named ANTP 2 pattern, (positions 46–55; Fig. 1) are present in all human HOXL and NKL genes and never present in human PRD homeodomains.

The pattern W [FY] [KQESR] [NK] [HQRKY] [RW] (positions 48–53; Fig. 1), named ANTP-PRD pattern, is found in all human PRD, NKL and HOXL homeodomains.

### 2) Generality of the amino acid patterns found

In order to test the generality of the amino acid patterns derived in the previous section, we used the 356 homeodomain sequences classified by Holland et al. [6], which include the 140 human sequences used above. These sequences include genes classified as HOXL, NKL and PRD, plus other homeobox gene classes (LIM, POU, HNF, SINE, TALE, CUT, PROS, ZF, CERS). It should be noted, that in these analyses, the difficult to classify genes that were excluded above were now included, and these were classified as tentatively suggested by Holland et al. [6]. Using amino acid sequences, a neighbour-joining tree (using pair-wise deletion, as implemented in the MEGA software; [17]) was built with the 356 sequences, plus 918 homeodomain sequences from a variety of animal species. The latter 918 sequences were obtained from the file PS50071 available at PROSITE [10] after removing all non-animal sequences from the file. Sequences from clusters that are supported by a bootstrap value of 80% or higher, and that include at least one sequence that has been classified by Holland et al. [6], as belonging to a given class or subclass, were classified as belonging to that class or subclass. Using this phylogenetic argument 202 sequences could be classified as HOXL, 204 as NKL, 200 as PRD, 66 as LIM, 83 as POU, 15 as HNF, 23 as SINE, 66 as TALE, 20 as CUT, 3 as PROS, 94 as ZF, and 10 as CERS. Nevertheless, 288 sequences remained unclassified.

Table 1 shows that HOXL 1 pattern is only found in genes classified as HOXL and thus is highly specific. It is also highly representative, because 96% of the HOXL sequences (194/202) used could be classified as such using this pattern. Therefore, the requirement to use one of the amino acid combinations implied by this pattern is a derived feature that appeared in the HOXL lineage early in animal evolution. The HOXL sequences that do not show the expected pattern are: *Drosophila melanogaster *AbdB, *Drosophila melanogaster *btn, *Strigamia maritima *Hox3b, *Gallus gallus *HMD2 (PROSITE annotation), *Danio rerio *HXABA (PROSITE annotation), and *Salmo salar *HXB2 (PROSITE annotation). These sequences do not form a closely related subgroup of sequences. For instance, the *Drosophila melanogaster Abd-B *and *btn *genes belong to two different HOXL gene families. Moreover, other genes belonging to these families do show the HOXL 1 amino acid pattern. The *Fugu rubripes *HXDBB (PROSITE annotation) and the *Gallus gallus *HXB8 (PROSITE annotation) sequences are incomplete, and thus it is not possible to determine whether they show this amino acid pattern. In addition to the 194 known HOXL genes showing the HOXL 1 pattern, 178/288 'unclassified' sequences also had this pattern (Table 1). Since the HOXL 1 pattern is very specific to the HOXL subclass, it is very likely that the 178 unclassified sequences that show this pattern are HOXL sequences.

**Table 1:** Number of sequences from each homeobox gene class or subclass showing a given amino acid pattern.

Pattern	HoxL (202)	NKL (204)	PRD (200)	LIM (66)	POU (83)	HNF (15)	SINE (23)	TALE (66)	CUT (20)	PROS (3)	ZF (94)	CERS (10)	Uncl. (288)
HoxL 1	194	0	0	0	0	0	0	0	0	0	0	0	178
HoxL 2	199	1	16	0	38	0	0	0	0	0	0	0	190
NKL	3	116	0	0	0	0	7	0	0	0	0	0	52
PRD	0	0	172	0	0	0	0	0	0	0	0	0	14
ANTP 1	197	199	1	1	7	12	0	11	9	0	25	2	251
ANTP 2	197	139	0	0	0	0	0	0	0	0	0	0	242
ANTP-PRD	200	200	190	52	0	0	23	0	0	0	0	0	275

Uncl. – unclassified sequences. The total number of sequences in each class is shown in parentheses.

The HOXL 2 pattern is not as specific as the HOXL 1 pattern, since it is observed in about 46% of the genes belonging to the POU class and in a few NKL (0.5%) and PRD (8%) genes. All 16 PRD genes showing the HOXL 2 pattern belong to the Pax4/6 gene family, thus it is likely that this is a case of convergent evolution. The human POU homeodomains showing HOXL 2 pattern are POU1F1, POU3F1, POU3F2, POU3F3, and POU3F4. The latter four genes are closely related, although the POU1F1 gene is distantly related to those genes [6]. Nevertheless, this is also likely a case of convergent evolution, since the alternative hypothesis (that the need to use one of the amino acid combinations implied by the HOXL pattern 2 is an ancestral feature) implies many independent losses. Therefore we argue that the necessity of using the amino acid combinations implied by HOXL 2 pattern is a derived feature that appeared early in the HOXL lineage. Only three sequences (1.5%) classified as HOXL using a phylogenetic argument, do not show the HOXL 2 pattern, namely: *Drosophila melanogaster *exex (the *Drosophila Mnx1 *orthologue; [18]), human MNX1 and mouse Mnx1 (PROSITE annotation). *Mnx1 *genes are peculiar in other ways. For instance, they show both the NKL and HOXL 1 pattern (see below).

The NKL pattern is highly specific but not widely representative of all NKL genes. The pattern is found in 57% of the sequences classified as belonging to the NKL class (116/204; Table 1), but in only ten other classified genes (three HOXL and seven SINE). In addition, 52 of the 288 phylogenetically 'unclassified' genes show the NKL pattern, and of these 36 have already been classified as belonging to the NKL class by PROSITE (data not shown). The three HOXL genes with the NKL pattern are the three *Mnx1 *genes (from *Drosophila melanogaster*, *Mus musculus*, and *Homo sapiens*). *Mnx1 *genes also show the HOXL 1 pattern that is highly specific for HOXL genes. Thus, it is unlikely that *Mnx1 *genes have been misclassified. Therefore, the presence of the NKL pattern in *Mnx1 *sequences may be the result of convergent evolution.

The seven SINE sequences where the NKL pattern is found are SIX3 from *Oryzias latipes*, *Gallus gallus*, *Mus musculus *and *Homo sapiens *and SIX6 from *Gallus gallus*, *Mus musculus *and *Homo sapiens*. All *SIX *human genes cluster together with a high bootstrap value (97%; [6]). Nevertheless, the NKL pattern is only observed in the *SIX3 *and *SIX6 *genes, two closely related genes. It is thus, likely a case of convergent evolution. The requirement to use one of the amino acid combinations implied by the NKL pattern is a derived feature that appeared likely early in the NKL lineage.

Most of the classified sequences where the NKL pattern is not found have been annotated by Holland et al. [6], and PROSITE as belonging to the NANOG, NOTO, VENTX, EN, DLX, and BARX, families. The EN, DLX and NOTO genes are difficult to classify. Thus, information on these genes was not used to derive the NKL amino acid pattern.

The PRD pattern is highly specific, being found only in sequences classified as PRD, and is also highly representative being found in 86% of the sequences classified as PRD. The PRD genes that do not show this pattern are *D. melanogaster OdsH*, *otp*, *PHDP*, *Ptx1*, *IP09201*, HOPX genes from *Danio rerio*, *Homo sapiens*, *Bos taurus*, *Rattus norvegicus*, *Mus musculus*, *Sus scrofa*, and *Gallus gallus *(PROSITE annotation), human *PAX2*, *PAX5 *and *PAX8 *(which have partial homeobox sequences), *Hydra vulgaris Dmbx*, OTP genes from *Heliocidaris erythrogramma*, *Heliocidaris tuberculata*, *Lytechinus variegates*, *Paracentrotus lividus*, and *Saccoglossus kowalevskii *(PROSITE annotation), *OTX from Strongylocentrotus purpuratus *(PROSITE annotation), *ALX *from *Strongylocentrotus purpuratus *(PROSITE annotation), *ANF *genes (PROSITE annotation) from *Gallus gallus*, and *Xenopus laevis *(two genes), and the *PROP *gene from *Canis familiaris *(PROSITE annotation). The PRD sequences that do not show this pattern are not related in any particular way. Therefore, the absence of this pattern in these sequences is likely the result of several independent losses. Therefore, it seems likely that the requirement to use one of the amino acid combinations implied by the PRD pattern is a derived feature that appeared early in the evolution of PRD genes.

Since the PRD-specific pattern is highly specific, it is likely that the 14 unclassified sequences that show this pattern are also PRD sequences. According to PROSITE classification these genes are *Gsc *from *Danio rerio*, *Xenopus laevis *(two genes), *Gallus gallus*, *Mus musculus*, *Saguinus labiatus*, *Gorilla gorilla*, *Pongo pygmaeus*, *Pan paniscus*, and *Pan troglodytes*, *ALX4 *from *Mus musculus *and *Bos taurus*, and *UNC-4 *and *ceh-36 *genes from *Caenorhabditis elegans *(PROSITE classification). According to PROSITE, 13 of these genes belong to the PRD class; the status of the *ceh-36 *gene is unknown.

The ANTP 1 pattern is found in most HOXL (98%) and NKL (98%) gene sequences and is almost absent in PRD (0.5%) gene sequences. Nevertheless, such a pattern is also found in sequences from genes belonging to all other classes except the PROS class (Table 1). It should, however, be noted that the sample size of PROS genes is very small (only three sequences). The ANTP sequences that do not show this pattern are *Drosophila melanogaster eve*, *lbe*, and *lbl*, *Homo sapiens NOTO*, *Heterodontus francisci Evx2 *(PROSITE annotation), *Gallus gallus Hme1 *(PROSITE annotation), *Fugu rubripes hxdbb*, and *Caenorhabditis elegans vab7 and hm31 *(PROSITE annotation). The *HXB8 *gene from *Gallus gallus *(PROSITE annotation) is incomplete, and thus it is not possible to determine whether it shows this pattern. The HOPX sequence (PROSITE annotation) from *Danio rerio *is the only PRD sequence that shows ANTP 1 pattern.

Although the ANTP 1 pattern is short (only three amino acid positions long), the broad distribution indicates that, very likely, it is not the result of convergent evolution. It is more likely that the ability to use the amino acid combinations implied by this pattern is an ancestral feature of homeobox containing genes. For some likely functional reason, ANTP genes have retained such a pattern, in contrast with PRD genes (the outgroup to ANTP genes) where only 0.5% of all sequences show this pattern.

The ANTP 2 pattern is found in most HOXL (98%) but in only 68% of the NKL sequences. This pattern is not found in sequences from other classes. It is thus, highly specific, although less representative across the NKL subclass of ANTP class genes. The HOXL genes that do not show this pattern are (PROSITE annotations) *Hmd2 *from *Gallus gallus*, *hxaba *from *Danio rerio*, *hxb2 *from *Salmo salar *and *hxbb *from *Fugu rubripes*. These sequences are not related in any particular way. It is not possible to determine whether the *Hxb8 *gene from *Gallus gallus *(PROSITE annotation) shows this pattern, since this is a partial sequence. NKL sequences not showing the ANTP 2 pattern belong to the Dlx (distalless), En (engrailed) and Noto gene families that have been previously found to be difficult to classify. Hence, information on these genes was not used to derive the ANTP 2 pattern. Once again, genes from these families stand out as oddities (it should be noted that they also do not show the NKL pattern as well; see above).

The ANTP-PRD pattern identifies most HOXL (99%), NKL (98%) and PRD (95%) sequences used. Nevertheless, it is also present in all SINE sequences and in 79% of the LIM sequences. The 16 ANTP plus PRD sequences that do not show the expected pattern are the *HOPX *genes from *Homo sapiens*, *Mus musculus*, *Rattus norvegicus*, *Sus scrofa*, *Bos taurus*, *Gallus gallus*, and *Danio rerio*, the *HM31 *gene from *Caenorhabditis elegans*, and the *Artemia sanfranciscana HMEN *gene. It should be noted that *HOPX *are difficult to classify, that in the latest revision [6] were classified as PRD genes, and these were not used to derive the amino acid patterns being tested. The *PAX2*, *PAX5 *and *PAX8 *sequences from *Homo sapiens*, as well as the *HXB8 *gene from *Gallus gallus*, the *HXDBB *gene from *Fugu rubripes*, and the *DLX2 *and *DLX4 *genes from *Eleutherodactylus coqui*, are partial, thus it is not possible to determine whether they show the ANTP-PRD amino acid pattern.

### 3) Further characterization of NKL genes

Given the failure to identify about 43% of the classified NKL sequences using the NKL pattern, we performed additional analyses to see whether the NKL pattern could be refined to accommodate the members of the NANOG, NOTO, VENTX, EN, DLX, and BARX, families. The results are shown in Table 2. When the NKL and refined patterns are considered, 98% of all NKL sequences could be classified as such. It should be noted that these refined NKL patterns are almost completely absent in the HOXL, PRD, LIM, POU, HNF, SINE, TALE, CUT, PROS, ZF and CERS classes. The 11 HOXL sequences that contain the EN pattern but not the NKL pattern all belong to the Hox1 gene family. This happens because the EN pattern allows for an asparagine at position 41. Five out of the six genes that show the NANOG pattern but not the NKL pattern belong to the HOXL *Gsx *gene family. These sequences are found because the pattern allows for a Lysine at position 43. The 8 HOXL sequences that show a hit when using the VENTX pattern but not the NKL pattern all belong to the *Gbx *family. This happens because a Valine is now allowed at position 43. The three CUT genes that show the EN and VENTX patterns are the *Homo sapiens ONECUT1 *gene and the orthologues in *Mus musculus *and *Rattus norvegicus*. These sequences show a hit because both patterns allow for an Isoleucine at position 45.

**Table 2:** Number of sequences showing only the refined NKL amino acid patterns.

Pattern	HoxL (202)	NKL (88)	PRD (200)	LIM (66)	POU (83)	HNF (15)	SINE (23)	TALE (66)	CUT (20)	PROS (3)	ZF (94)	CERS (10)	Uncl. (288)
DLX-BARX	0	40	0	0	0	0	0	0	0	0	0	0	2
EN	11	28	0	0	0	0	1	0	3	0	0	0	21
NOTO	0	1	0	0	0	0	0	0	0	0	0	0	2
NANOG	6	8	0	0	0	0	0	0	0	0	0	0	1
VENTX	8	6	0	0	0	0	0	0	3	0	0	0	6

Uncl. – unclassified sequences. The total number of sequences in each class is shown in parentheses.

DLX-BARX – [AKST] [QDENPS] [LAST] [Q] [V]; EN – [AKSTN] [DENPS] [LAST] [Q] [VI];

NOTO – [AKST] [DENPS] [LASTN] [Q] [V]; NANOG – [AKST] [DENPSY] [LASTK] [Q] [V];

VENTX – [AKST] [DENPS] [LASTV] [Q] [VI]

### 4) Non-bilaterian homeobox sequences

Only a few non-bilaterian homeobox sequences (those listed in [6]) are contained in the data sets used. Therefore, we collected from the NCBI database a set of 251 non-redundant non-bilaterian homeobox sequences that encompass the regions where the amino acid patterns here reported are located, and that showed the ANTP-PRD pattern derived above (see Appendix). Most bilaterian ANTP and PRD sequences show this ANTP-PRD pattern (Table 1); furthermore, this pattern is only found in ANTP, PRD, LIM and SINE sequences (Table 1). Therefore, by imposing the presence of the ANTP-PRD pattern we hoped to enrich the data set for non-bilaterian ANTP and PRD sequences. Table 3 summarizes the results. As expected, of the retrieved gene sequences only one seems to belong to gene classes other than ANTP, PRD, LIM and SINE.

**Table 3:** Amino acid pattern presence in non-bilaterian sequences.

Patterns other than ANTP-PRD	Expected pattern for	HoxL	NKL	PRD	Demox	LIM	SINE	TALE	Uncl.
ANTP1; ANTP2; HOXL1; HOXL2	HOXL								13
ANTP1; ANTP2; HOXL1; NKL	Mnx1		1						
ANTP1; ANTP2; NKL	NKL		22						12
ANTP1; NKL	NKL*		4	1			1		7
ANTP1; ANTP2; EN	EN (NKL)								5
ANTP1; ANTP2; NOTO	NOTO (NKL)		3						4
ANTP1; ANTP2; VENTX	VENTX (NKL)		1						
ANTP1; ANTP2	Demox		1		3				14
PRD	PRD			24					2
Other pattern combinations	?	1	20	33		5	14	1	59

Only those non-bilaterian sequences that encompass the regions where the amino acid patterns here described are located, and that showed the ANTP-PRD pattern were used. In total 251 non-redundant sequences were used (gi numbers are listed in the appendix). Cases where non-bilaterian genes are clearly misidentified when using amino acid patterns are shown underlined (see text for details). Uncl. – unclassified sequences.

* this signature can also be considered characteristic of NKL genes since only 68% of bilaterian NKL sequences show the ANTP2 pattern

Most non-bilaterian genes showing the full NKL or PRD signature have already been classified as such. Furthermore, the ANTP Demox class found in Demospongiae and apparently absent in all other animals [19], is characterized by the presence of the ANTP-PRD, and ANTP 1 and 2 patterns, as expected for ANTP genes that do not belong to the HOXL or NKL lineage (Table 3). Although there are no true Hox genes in sponges [20], 13 sequences show the full HOXL signature. Interestingly, all such sequences are non-annotated, thus they must be hard to classify gene sequences. One *Ephydatia fluviatilis *gene has been labeled as the *Msx *(NKL) gene. Our pattern analyses show that the corresponding sequence shows a pattern exclusively found in the *Mnx1 *(HOXL) gene family, thus, also suggesting that non-bilaterian sequences with full signatures may be wrongly identified. About 53% of all non-bilaterian sequences used did not show an easily recognizable signature. We conclude that the reported amino acid patterns must be cautiously used when applied to sequences from non-bilaterian animals.

### 5) Identification of single key amino acid changes in bilaterian PRD, NKL and HOXL genes

Key amino acid changes that occurred during the evolution of homeobox containing genes may be revealed as changes affecting a single amino acid position. Thus, we compared, at each amino acid position, the large HOXL (202 sequences), NKL (204 sequences) and PRD (200 sequences) data sets described above, for the following chemical properties: size, charge, polarity, hydrophobicity, aromaticity, and aliphapaty. For completeness, the 178, 52 and 14 sequences that could not be classified using a phylogenetic argument but that show amino acid patterns typical of HOXL, NKL and PRD genes were also used.

Chemical properties observed in more than 99% of the sequences belonging to one class, and observed in less than three-quarters of sequences belonging to another class are shown in Tables 4, 5, and 6. Rules are described relative to the inferred common ancestor situation of the two categories being compared. For instance, when comparing PRD and ANTP class genes the rule "not negatively charged at position 27" means that it was inferred (by comparison with LIM genes) that the common ancestor to these two gene classes could use a negatively charged amino acid at position 27; in this example, ANTP genes subsequently almost completely lost the ability to use a negatively charged amino acid, but 94% of PRD genes retained the use of such an amino acid at this position (Table 4). An almost complete change in amino acid usage regarding polarity and hydrophobicity is also observed for amino acid position 30. Furthermore, about 30% of the PRD genes show a charged amino acid at position 50. HOXL and NKL genes never use charged amino acids at this position, and this is also the case for LIM genes, here used as an outgroup to ANTP and PRD genes. Therefore, the possibility of using a charged amino acid at position 50 seems to be a derived feature that appeared in the PRD lineage. This amino acid position has been previously used to sub-classify PRD genes into three categories [21]. A charged amino acid is found in sequences from *Caenorhabditis elegans*, *Strongylocentrotus purpuratus*, and *Hydra vulgaris*, among others, thus this switch is an event that happened early in the evolution of PRD genes.

**Table 4:** Frequency of genes following amino acid rules specific for the PRD or ANTP classes.

Position	27		29		30		30		50	
Rules	Not Ne		Not A		Not (LP, NP)		Not (LH, VH)		(P,Ne)	
Dataset	Phy	Unc	Phy	Unc	Phy	Unc	Phy	Unc	Phy	Unc
PRD	0.060 (200)	0.000 (14)	0.305 (200)	0.714 (14)	0.020 (200)	0.071 (14)	0.020 (200)	0.071 (14)	0.315 (197)	0.786 (14)
(HOXL and NKL)	0.998 (406)	1.000 (230)	1.000 (406)	1.000 (230)	0.998 (406)	1.000 (230)	0.998 (406)	1.000 (230)	0.000 (404)	0.000 (230)

In brackets is indicated the total number of sequences analysed.

Phy – set of sequences classified using a phylogenetic approach

Unc – set of sequences not classified using a phylogenetic approach. These sequences, nevertheless, show amino acid patterns typical of PRD, HOXL and NKL genes. Ne – negatively charged amino acids; P – positively charged amino acids; A -aromatic amino acids; LP – Less-polar amino acids; NP – Non-polar amino acids; LH – less hydrophobic amino acids; VH – very hydrophobic amino acids; S- small amino acids; T – tinny amino acids; Al – Alyphatic amino acids.

**Table 5:** Frequency of genes following amino acid rules specific for the HOXL or NKL sub-classes (positions 1 to 30).

Position	14		15		15		15		28	
Rules	Not A		Not (LH, VH)		Not (LP, NP)		Not (S, T)		Not (S, T)	
Dataset	Phy	Unc	Phy	Unc	Phy	Unc	Phy	Unc	Phy	Unc
HoxL	0.995 (202)	1.000 (177)	1.000 (202)	1.000 (177)	1.000 (202)	1.000 (177)	1.000 (202)	1.000 (177)	0.990 (202)	1.000 (178)
NKL	0.709 (203)	0.808 (52)	0.588 (204)	0.346 (52)	0.598 (204)	0.346 (52)	0.637 (204)	0.346 (52)	0.500 (204)	0.673 (52)

In brackets is indicated the total number of sequences analysed.

Legend as in Table 4

**Table 6:** Frequency of genes following amino acid rules specific for the HOXL or NKL sub-classes (positions 31 to 60).

Position	33		33		47		54	
Rules	Not (LH, VH)		Not (NP, LP)		Not (Non-Al)		Not LP	
Dataset	Phy	Unc	Phy	Unc	Phy	Unc	Phy	Unc
HoxL	1.000 (202)	1.000 (178)	1.000 (202)	1.000 (178)	1.000 (201)	1.000 (178)	1.000 (200)	1.000 (178)
NKL	0.716 (204)	0.865 (52)	0.701 (204)	0.865 (52)	0.730 (204)	0.981 (52)	0.431 (202)	0.731 (52)

In brackets is indicated the total number of sequences analysed.

Legend as in Table 4

Genes belonging to the HOXL lineage show derived constraints at amino acid positions 14, 15, 28, 33, 47 and 54, relative to the inferred ancestral state for ANTP class genes (the PRD data set was used as an outgroup; Table 4). Only three sequences classified as HOXL do not follow the general pattern for these genes, namely, the *HMA2 *gene (PROSITE annotation) from *Helobdella triserialis *that shows an aromatic amino acid at position 14, and the *HXB8 *and *PAL1 *genes (PROSITE annotation) from *Sus scrofa *and *Caenorhabditis elegans*, respectively, that show small amino acids at position 28. No derived single amino acid constraints were found for the NKL lineage.

## Discussion

The amino acid patterns and single key amino acid changes here identified shed light on some of the major likely functional changes that occurred during the evolution of the HOXL, NKL and PRD genes. Our results show that important amino acid changes happened very early in the evolution of these genes, and thus it is possible to identify an archetype for bilaterian PRD, ANTP, HOXL and NKL genes. As with every generalization, however, some genes do not fit the archetype. Experimental studies are now needed in order to understand why the archetypes possess such chemical properties. We propose the following archetypes for homeodomain amino acid sequences showing the ANTP-PRD pattern (this pattern is present in 97% of all bilaterian ANTP and PRD sequences).

PRD genes show the L [EINQRV] [^DGHMPTVWY] [^CDGKMNPQR] [FL] [^CFILPTWY] [AEFHKQRV] [ADEGKNSTW] [CHKMPQR] [FHY]P (positions 16–26) PRD pattern, use a negatively charged amino acid at position 27 (DE) and a less polar or non-polar amino acid (^RKDENQ) at position 30. About 80% of all animal bilaterian PRD genes follow this description.

ANTP (Demox, HOXL and NKL) genes show the [HQ] [IV] [AKLT] (positions 44–46) ANTP 1 pattern. In contrast to PRD genes, ANTP genes do not use a negatively charged amino acid at position 27 (DE). Furthermore, at position 30, 99.8% of all bilaterian ANTP genes use a polar amino acid (RKDENQ). 97% of all ANTP bilaterian genes follow this description (data not shown).

HOXL genes are characterized by the presence of the HOXL 1 pattern. 96% of all bilaterian HOXL genes show the [KT] [IV]WFQNRR [AMV]K [DEHKLMQWY] [KR] [KR] (positions 46–58) HOXL 1 pattern.

NKL genes show the NKL pattern (positions 41–45) or a NKL derived pattern. About 98% of the bilaterian NKL genes follow this pattern. If the NKL pattern is generalized to include the derived NKL patterns then many hits are observed in other homeodomain classes (data not shown). This is why several NKL-related patterns are reported.

The above definitions suggest that the region in between amino acid positions 16–30 and 44–58 are the most important for function specificity of genes belonging to different classes and sub-classes. The former region corresponds to the end of Helix 1, the inter Helix 1–2 region and to the first positions of Helix 2 [9]. The region in between amino acid positions 44–58 correspond mainly to helix 3. This helix, also called recognition helix is essential for successful and specific DNA binding [9].

The situation observed for NKL genes suggest that specific amino acid patterns may exist for groups of genes within major classes, as previously suggested [9]. It also indicates that not all motifs implied by an amino acid pattern occur in a given homeodomain class. On the other hand, the situation observed for non-bilaterian vs bilaterian genes suggests that we may have failed to identify all relevant chemical changes. For instance, it is conceivable that important amino acid changes are observed when the protostome and the deuterostome lineages are compared; such distinctions were not addressed in this work because we grouped genes from a given homeodomain class together, irrespective of the organism.

It is tempting to use these features to classify homeobox containing genes. Nevertheless, given the very old age of the gene families considered, the possibility of convergent evolution must be considered when analyzing a given amino acid sequence. Therefore, for the purpose of gene classification, the features here described should not be decisive but rather used as additional evidence. It should be noted, that the reconstruction of gene genealogies is a hard problem, namely when proteins belonging to the same family share some of the same protein interaction partners, thus facing a similar selective environment (Campos et al. 2004). Any additional piece of evidence that may shed light on the correct classification of genes should therefore be used.

## Conclusion

In this work we report a method for the fast retrieval of bilaterian ANTP and PRD sequences. Given the availability of a sufficiently large curated data set this method can be applied to any group of proteins. Furthermore, we report some of the main amino acid changes that occurred early in the HOXL, NKL and PRD lineages. These features can be used for the classification of gene sequences, although, as shown, convergent evolution must be considered as an explanation for the presence of a given pattern in a sequence. The possibility that the region of the protein that allows distinguishing the different classes, also allows the distinction of different families within classes, has important practical and evolutionary consequences and must be explored in more detail.

## Authors' contributions

NAF, JV and CPV conceived the design of the study. NAF developed the software needed for the large-scale analyses, while JV and CPV collected, aligned the sequence data, and performed the phylogenetic analyses. JV drafted the manuscript. All authors participated in the results discussion and helped writing the final version of the manuscript. All authors read and approved the final manuscript.

## Appendix

Gi numbers of the 251 non-bilaterian non-redundant sequences used for the analysis presented in Table 3. All sequences show the ANTP-PRD amino acid pattern.

### Sequences showing patterns ANTP-PRD, ANTP1, ANTP2, HOXL1 and HOXL2

*Nematostella vectensis*: 32263856; 32263873; 74039490; 74039492; 82621559; 82621587; 82621611; 82621663; 83356315; 156363224; 156363226; 156387518; 156397205

### Sequences showing patterns ANTP-PRD, ANTP1, ANTP2, HOXL1 and NKL

*Ephydatia fluviatilis*: 438584

### Sequences showing patterns ANTP-PRD, ANTP1, ANTP2 and NKL

*Acropora millepora*: 117581722; 117581724; 117581726; 117581728; *Anemonia erythraea*: 158936936; *Ephydatia fluviatilis*: 438585; 510584; *Halichondria bowerbanki*: 33641771; *Nematostella vectensis*: 32816231; 78190373; 82621509; 82621525; 82621533; 82621555; 82621557; 82621585; 82621591; 82621609; 82621625; 82621657; 82621665; 82621667; 110339021; 110339029; 110339121; 156353243; 156363798; 156367335; 156372762; 156397943; 168693291;*Suberites domuncula*: 34786940; 49659003; *Sycon raphanus*: 11066243

### Sequences showing patterns ANTP-PRD, ANTP1 and NKL

*Anthopleura japonica*: 144369330; 144369363; *Hydra vulgaris*: 2331219; 7635735; *Nematostella vectensis*: 82570553; 82621543; 82621677; 110339077; 110339133; 156376845; 156402818; 156406963; 156407174

### Sequences showing patterns ANTP-PRD, ANTP1, ANTP2 and EN

*Nematostella vectensis*: 82621563; 82621631; 82621655; 156366927; 156402173

### Sequences showing patterns ANTP-PRD, ANTP1, ANTP2 and NOTO

*Nematostella vectensis*: 82621579; 82621647; 82621651; 110339063; 156388033; 156400908; 156407394

### Sequences showing patterns ANTP-PRD, ANTP1, ANTP2 and VENTX

*Nematostella vectensis*: 110339027

### Sequences showing patterns ANTP-PRD, ANTP1 and ANTP2

*Baikalospongia intermédia*: 62238212; *Ephydatia fluviatilis*: 1438870; *Ephydatia muelleri*: 3184520; *Hydra vulgaris*: 7635740; *Nematostella vectensis*: 82621517; 82621615; 82621619; 82621623; 82621635; 82621653; 156375827; 156375891; 156376334; 156401296; 156402692; *Podocoryne carnea*: 28188799; *Potamolepis sp*.: 62238208; *Suberites domuncula*: 49659001

### Sequences showing patterns ANTP-PRD and PRD

*Anthopleura japónica*: 144369334; *Cladonema californicum*: 9652040; *Hydra vulgaris*: 3021450; 3021452; 6503072; *Nematostella vectensis*: 38569883; 78190377; 82395396; 82395402; 82395404; 82395406; 82395408; 82395412; 82395414; 82570541; 82570557; 82570559; 82570563; 110339183; 110339213; 110339215; 110339225; 113120207; 156377162; 156615306;*Tripedalia cystophora*: 33391193

### Sequences showing other pattern combinations

*Acropora formosa*: 228960; *Acropora millepora*: 7335704; 7595811; 7595813; 13506878; 117581730; 117581732;*Anthopleura japónica*: 144369323; 144369326; 144369366; *Aurelia aurita*: 50841484; *Cassiopea xamachana*: 4894653; 4894655; 4894659; *Cladonema radiatum*: 47155918; 47155920; 47155922; *Eleutheria dichotoma*: 1147626; 91982983; 91982989; *Hydra littoralis*: 2102728; *Hydra magnipapillata*: 630481; 630482; 89242120; 144369369; 144369375; *Hydra viridis*: 7120; 7124; 7130; 83763566; *Hydra vulgaris*: 4433647; 4838455; 7635731; 7635733; 7635737; 7635742; 9945022; *Hydractinia symbiolongicarpus*: 2980868; 83272159; *Nematostella vectensis*: 5081328; 32816237; 74039494; 78190375; 82395398; 82395410; 82570519; 82570521; 82570527; 82570529; 82570537; 82570539; 82570545; 82570549; 82570555; 82570561; 82621507; 82621511; 82621513; 82621515; 82621519; 82621523; 82621527; 82621531; 82621535; 82621539; 82621541; 82621547; 82621549; 82621551; 82621565; 82621567; 82621571; 82621573; 82621575; 82621589; 82621595; 82621601; 82621603; 82621605; 82621613; 82621621; 82621627; 82621629; 82621633; 82621637; 82621643; 82621645; 82621671; 82621675; 83356317; 99030986; 110338989; 110339023; 110339061; 110339099; 110339101; 110339115; 110339171; 110339191; 110339211; 110339247; 113120203; 156356964; 156358580; 156361301; 156361303; 156364678; 156368221; 156371265; 156371439; 156372678; 156374167; 156377158; 156377160; 156377164; 156389076; 156390886; 156393340; 156395806; 156396978; 156398319; 156407912; *Podocoryne carnea*: 976094; 6118056; 6465862; 7649930; 9964019; 47155914; 62002543; *Sarsia sp*.: 9988771; *Scolionema suvaense*: 158936914; *Suberites domuncula*: 49659005; *Trichoplax adhaerens*: 38565482
